# Incidence, risk factors and mortality of tuberculosis in Danish HIV patients 1995-2007

**DOI:** 10.1186/1471-2466-11-26

**Published:** 2011-05-23

**Authors:** Gry A Taarnhøj, Frederik N Engsig, Pernille Ravn, Isik S Johansen, Carsten S Larsen, Birgit Røge, Aase B Andersen, Niels Obel

**Affiliations:** 1 Department of Infectious Diseases, Copenhagen University Hospital, Rigshospitalet, Blegdamsvej 9, 2100 Copenhagen Ø, Denmark; 2 Department of Infectious Diseases, Copenhagen University Hospital, Herlev Hospital, Herlev Ringvej 75, 2730 Herlev, Denmark; 3 Department of Infectious Diseases, Copenhagen University Hospital, Hvidovre Hospital, Kettegård Allé 30, 2650 Hvidovre Denmark; 4 Department of Infectious Diseases, Aarhus University Hospital, Skejby Sygehus, Brendstrupgårdsvej 100, 8200 Århus N, Denmark; 5 Department of Infectious Diseases, Odense University Hospital, Sønderboulevard 29, 5000 Odense, Denmark

## Abstract

**Background:**

Human Immunodeficiency Virus (HIV) infection predisposes to tuberculosis (TB). We described incidence, risk factors and prognosis of TB in HIV-1 infected patients during pre (1995-1996), early (1997-1999), and late Highly Active Antiretroviral Therapy (HAART) (2000-2007) periods.

**Methods:**

We included patients from a population-based, multicenter, nationwide cohort. We calculated incidence rates (IRs) and mortality rates (MRs). Cox's regression analysis was used to estimate risk factors for TB infection with HAART initiation included as time updated variable. Kaplan-Meier was used to estimate mortality after TB.

**Results:**

Among 2,668 patients identified, 120 patients developed TB during the follow-up period. The overall IR was 8.2 cases of TB/1,000 person-years of follow-up (PYR). IRs decreased during the pre-, early and late-HAART periods (37.1/1000 PYR, 12.9/1000 PYR and 6.5/1000 PYR respectively). African and Asian origin, low CD4 cell count and heterosexual and injection drug user route of HIV transmission were risk factors for TB and start of HAART reduced the risk substantially. The overall MR in TB patients was 34.4 deaths per 1,000 PYR (95% Confidence Interval: 22.0-54.0) and was highest in the first two years after the diagnosis of TB.

**Conclusions:**

Incidence of TB still associated with conventional risk factors as country of birth, low CD4 count and route of HIV infection while HAART reduces the risk substantially. The mortality in this patient population is high in the first two years after TB diagnosis.

## Background

The prognosis for Human Immunodeficiency Virus (HIV) infected patients has changed dramatically after the introduction of Highly Active Antiretroviral Therapy (HAART) [1]. In spite of the effect of HAART, tuberculosis (TB) is still one of the most frequently acquired immune-deficiency syndrome (AIDS) defining conditions worldwide [2]. Though effective, prevention of TB in HIV positive by tuberculin skin testing and targeted preventive treatments is of limited clinical success [3,4]. In contrast, implementation of HAART has been the best preventive measure and incidence rates of TB co-infection have from Western World settings been reported to decrease from the pre- to late-HAART period. A European study reported incidence rates before September 1995 of 0.8/100 person-years of follow-up (PYR) decreasing to 0.3/100 PYR after March 1997 [5]. The identification of risk factors for development of TB is crucial for the design of strategies to control the TB epidemic in the HIV-infected population. Previously conducted studies in developed countries all present significant associations between both immigration from high-endemic TB regions and low CD4 cell counts and increased risk of developing active TB [2,6-8].

To our knowledge, no nationwide population-based studies have been conducted on the incidence, risk factors and prognosis of HIV-associated TB during the HAART era. The objective of the present study was to determine the incidence of TB in the Danish HIV-infected population during the pre-HAART (1995-1996), early HAART (1997-1999), and late HAART (2000-2007) periods and risk factors for developing TB. We further aimed to describe mortality after TB diagnosis.

## Methods

### Study design

In the first part of the analysis, the study population was the Danish HIV population (see below) diagnosed with HIV in the period 1995-2007. In this population we estimated incidence and risk factors for being diagnosed with TB. In the second part of the study, the study population was the HIV patients diagnosed with TB and we determined mortality after diagnosis of TB.

### Setting

Denmark had a population of 5.5 million as of 31 December 2007 [9], with an estimated HIV prevalence of approximately 0.07% in the adult population [10]. Patients with HIV infection are treated in one of eight specialized medical centres in the country, where the patients are seen on an outpatient basis at intended intervals of 12 to 16 weeks. Antiretroviral treatment is provided free of charge to all HIV-infected residents of Denmark. The national criteria for initiating HAART have previously been described [11]. Directly observed treatment (DOT) is not uniformly performed at the centres and treatment for latent tuberculosis is used infrequently in Denmark. TB diagnostic services are centralised at the Mycobacteriology laboratory at Statens Serum Institut in Copenhagen. This is the only laboratory in Denmark performing culture analyses for TB ensuring that the diagnostic standard is uniform for all participating centres.

### Data sources

We used the unique 10-digit central person registration number (CPR number), assigned to all Danish citizens at birth or immigration, to track individuals in the following registries and to avoid multiple registrations.

The Danish HIV Cohort Study (DHCS) is a prospective, nationwide, population-based cohort study of all HIV-infected patients treated in Danish HIV clinics since 1 January 1995. Data are updated yearly and includes demographics, date of HIV infection, AIDS-defining events, date and reason of death, antiretroviral treatment, CD4 cell counts and HIV-RNA measurements. Study objects were identified from this registry as were date of HIV diagnosis, TB diagnosis, demographics, CD4 cell counts, viral loads and antiretroviral treatment.

The Danish Civil Registration System (DCRS) is a national registry of all Danish residents established in 1967 that contains demographic data and vital status of all Danish citizens. From this registry, we extracted date of emi- and immigration and date of death.

The Danish National Hospital Registry (DNHR) was initiated in 1977 and contains information on all patients discharged from Danish non-psychiatric hospitals including discharge diagnoses coded by the treating physician according to the International Classification of Diseases 10th revision (ICD-10) from 1994 and onwards. From this registry, we extracted data on admissions under all alcohol related diagnoses (F10.0 - F10.9 and K70.2 - K70.3).

The Danish Registry of Causes of Death (DRCD) contains information from all Danish death certificates since 1943 and registration is currently complete through 2006. Causes of death are coded according to ICD-10 from 1994 through 2006. The causes of death are registered by the treating physician on the death certificate as primary (immediate cause of death), secondary or tertiary cause of death. From this registry we extracted causes of death as recorded as the primary cause of death [12] and classified the causes of death into 3 categories: (1) related to TB, (2) not related to TB, but related to HIV infection, or (3) not related to HIV or TB.

### Study population

We included all patients > 15 years diagnosed with HIV-1 in Denmark in the period 1 January 1995 to 31 December 2007 and living in Denmark at the date of HIV diagnosis.

### Outcomes

In the first part of the study, outcome was time to first diagnosis of TB (pulmonary as well as extra pulmonary). Only TB cases diagnosed in the study period after HIV diagnosis or within three months prior to HIV diagnosis were included. A diagnosis of TB was defined as either (1) *definite*, being the finding of acid fast bacteria in sputum or body fluid/tissue by microscopy or PCR or culture positive for Mycobacterium tuberculosis or (2) *presumptive*, being clinical symptoms and/or paraclinical findings suggestive of TB prompting anti-TB treatment to be initiated and completed. In the second part of the study outcome was time from TB diagnosis to death.

### Statistics

Differences in characteristics between groups were evaluated by the χ^2^test and Fisher's exact test. Differences were considered significant if P < 0.05.

In the first part of the study, time was calculated from date of HIV diagnosis to date of TB diagnosis. Patients were censored at date of last clinical follow-up, date of death or 31 December 2007 which ever came first. Incidence rates (IRs) for TB diagnosis and 95% confidence intervals (95% CI) were calculated for the periods 1995-1996, 1997-1999, 2000-2007 corresponding to pre, early and late HAART periods. We also calculated IRs for TB for the following periods after HIV diagnosis: 0-1 year, 2-5 years and 6-10 years. Further, we used Cox regression model to calculate incidence rate ratios (IRRs) as estimates of the impact of risk factors on incidence of TB in the HIV infected population.

In the second part of the study, time was calculated from date of TB diagnosis to date of death, emigration or 31 December 2007, whichever came first. For patients diagnosed with TB within three months prior to HIV diagnosis, time was calculated from date of HIV diagnosis.

We used Kaplan-Meier analysis to construct survival curves.

The following covariates were included in the uni- and multivariate models: gender, age at HIV diagnosis (≤ 40 years vs. > 40 years), country of birth/region (Denmark vs. Europe vs. Africa vs., Asia vs. other), year of HIV diagnosis (1995-1996 vs. 1997-1999 vs. 2000-2007), CD4 cell count at HIV diagnosis in the first part of the study (≤ 200 cells/μL vs. > 200 cells/μL), CD4 cell count at TB diagnosis in the second part of the study (≤ 200 cells/μL vs. > 200 cells/μL), viral load at HIV diagnosis in the first part of the study and viral load at TB diagnosis in the second part of the study (≤ 5.0 log_10 _copies/mL vs. > 5.0 log_10 _copies/mL), route of HIV transmission (heterosexual vs. homosexual vs. injection drug user (IDU) vs. other) and alcohol abuse (previous inpatient admission under alcohol-related diagnoses vs. no previous inpatient admission under alcohol-related diagnoses). We included date of first HAART initiation as a time dependent variable.

The study was approved by the Danish Data Protection Agency. SPSS version 15.0 was used for data analysis.

## Results

From the Danish HIV Cohort Study we identified 2,668 patients diagnosed with HIV in the period 1995-2007 contributing with 14,711 person-years of follow up. The characteristics of the study population are shown in table 1. Due to HIV diagnosis prior to arrival in Denmark, 233 patients were excluded from the study of whom 6 developed TB after immigration to Denmark. In the study period, 120 patients met the criteria for a diagnosis of TB. Overall the incidence in the period 1995-2007 was estimated to 8.2/1,000 person-years of follow-up (PYR). Incidence rates decreased considerably from the pre- to the early- and late-HAART periods (table 2). The incidence of TB was highest in the first year after HIV diagnosis and stabilized in the following years (table 2).

**Table 1:** Characteristics of the HIV patients in the study population

Characteristics		N = 2,668
Emigrated during study period		90 (3%)
Died during study period		308 (12%)
Males		1,991 (75%)
Caucasians		1,944 (73%)
Country of birth		
	Denmark	1,843 (69%)
	Europe	165 (6%)
	Africa	412 (15%)
	Asia	166 (6%)
	Other	82 (3%)
Route of HIV transmission		
	Heterosexual	1,135 (43%)
	Homosexual	1,090 (41%)
	IDU	220 (8%)
	Other	223 (8%)
Median age at HIV diagnosis, years (IQR*)		36.9 (30.3-45.2)
Median CD4 cell count at HIV diagnosis, cells/μL (IQR*)**		290 (109.25-500.0)
Median HIV RNA at HIV diagnosis, log_10 _copies/mL (IQR*)***		4.8 (4.2-5.4)
Exposed to HAART during study period		1,987 (75%)
Diagnosed with alcohol abuse		145 (5%)

* IQR: Inter Quartile Range.

** 532 patients had missing CD4 cell count at time of HIV diagnosis.

*** 673 patients has missing viral load at time of HIV diagnosis.

**Table 2:** Incidence of TB stratified by year of HIV diagnosis

Period	Observation time PYR*	Number TB diagnoses	Incidence rate (TB/1000 PYR*)	95% CI**
1995-07	14710.5	120	8.2	6.8-9.8
1995-96	404.6	15	37.1	22.4-61.5
1997-99	1932.2	25	12.9	8.7-19.1
2000-07	12373.6	80	6.5	5.2-8.1

Incidence of TB stratified by years after HIV-diagnosis				

0-1 yr	2415.8	95	39.3	32.2-48.1
2-5 yrs	7175.8	17	2.4	1.5-3.8
6-10 yrs	4438.4	6	1.4	0.6-3.0

* PYR: person-years of follow-up.

** CI: Confidence Interval.

The characteristics of patients diagnosed with TB are shown in table 3. There were 120 patients (4.5%) diagnosed with TB among the 2,668 study participants, of whom 69 (57.5%) had pulmonary TB, 38 (31.7%) had extrapulmonary TB and 13 (10.8%) had both. At time of the TB diagnosis 69 (67.0%) of the patients had a CD4 cell count less than 200 cells/μL. Only 28 (23.3%) patients had started HAART prior to their TB diagnosis, while 92 (76.7%) were diagnosed with TB before start of HAART. Among the 92 patients, 29 (31.5%) were diagnosed with TB simultaneous with the HIV diagnosis, 15 (16.3%) were diagnosed with TB after the HIV diagnosis and had a CD4 cell count of more than 200 cells/μL and among the remaining 48 (52.2%) patients the median time from HIV diagnosis to TB diagnosis was 10.5 days (IQR; 6.0 - 30.5). We found no major differences in the characteristics of patients diagnosed with TB in the periods 1995-1996, 1997-1999 and 2000-2007.

**Table 3:** Characteristics of the HIV infected patients diagnosed with TB

Year of TB diagnosis		TB 1995-2007 N = 120	TB 1995-1996 N = 15	TB 1997-1999 N = 25	TB 2000-2007 N = 80	P value
Males		70 (58.3%)	11 (73.3%)	16 (64%)	43 (53.8%)	0.30
Median age, years (IQR*)		35.4 (30.8-42.5)	33.6 (31.2-38.5)	35.1 (29.6 - 45.5)	37.1 (30.8-42.9)	0.41
Birth country/region						0.80
	Denmark	45 (37.5%)	5 (33.3%)	11 (44.0%)	29 (36.3%)	
	Europe	4 (3.3%)	1 (6.7%)	1 (4.0%)	2 (2.5%)	
	Africa	45 (37.5%)	7 (46.7%)	10 (40.0%)	28 (35.0%)	
	Asia	22 (18.3%)	2 (13.3%)	2 (8.0%)	18 (22.5%)	
	Other	4 (3.3%)	0 (0.0%)	1 (4.0%)	3 (3.8%)	
Transmission of HIV						0.96
	Homosexual	18 (15.0%)	3 (20.0%)	4 (16.0%)	11 (13.8%)	
	Heterosexual	72 (60.0%)	9 (60.0%)	16 (64.0%)	47 (58.8%)	
	IDU	15 (12.5%)	2 (13.3%)	2 (8.0%)	11 (13.8%)	
	Other	15 (12.5%)	1 (6.7%)	3 (12.0%)	11 (13.8%)	
Diagnosis of alcohol abuse		13 (10.8%)	1 (6.7%)	2 (8.0%)	10 (12.5%)	0.38
Median HIV RNA at time of TB diagnosis, log_10 _copies/mL (IQR*)**		4.9 (3.6-5.9)	3.0 (2.7-3.9)	4.9 (4.2-5.9)	4.9 (3.6-5.9)	0.87
Median CD4cell count at time of TB diagnosis, cells/μL (IQR*)***		120 (50-280)	150 (8-175)	110 (42-295)	120 (60 - 362)	0.94
CD4 cell count < = 200 cells/μL at time of TB diagnosis***		69 (67.0%)	9 (81.8%)	14 (66.7%)	46 (64.8%)	0.54
Caucasians		43 (35.8%)	5 (33.3%)	11 (44.0%)	27 (33.8%)	0.67
HAART started before TB diagnosis		28 (23.3%)	1 (6.7%)	5 (20.0%)	22 (27.5%)	0.20
HAART during study period		104 (86.7%)	12 (80%)	23 (92.0%)	69 (86.3%)	0.55
Emigrated during study period		7 (5.8%)	1 (6.7%)	1 (4%)	5 (6.3%)	0.91
Dead during study period		19 (15.8%)	3 (20.0%)	6 (24.0%)	10 (12.5%)	0.35
Death within 2 yrs after HIV diagnosis		17 (14.2%)	3 (20.0%)	6 (24.0%)	8 (10.0%)	0.17
Median time from HIV to TB, months (IQR*)		0.6 (< 0.1-7.2)	0.6 (0.1-2.9)	0.4 (< 0.1 - 2.6)	0.8 (< 0.1-15.7)	0.30
Median time from TB to death, months (IQR*)		12.6 (0.6-17.8)	13.6 (0.0-58.8)	10.8 (0.5-41.6)	11.8 (1.7-17.7)	0.95

* IQR: Inter Quartile Range.

** 24 patients had missing viral load at time of TB diagnosis.

*** 17 patients had missing CD4 cell count at time of TB diagnosis.

In univariate analysis the following factors were associated with an increased risk of TB diagnosis: Male gender (IRR: 2.1; 95% CI: 1.5-3.0; p-value < 0.001), age < 40 years (IRR: 1.5; 95% CI: 1.0-2.3; p-value 0.036), country/region of birth (Africa (IRR: 4.7; 95% CI: 3.1-7.0; p-value < 0.001) and Asia (IRR: 5.9; 95% CI: 3.5-9.8; p-value < 0.001)) route of HIV transmission (heterosexual (IRR: 3.9; 95% CI: 2.3-6.5; p-value < 0.001), IDU (IRR: 4.2; 95% CI: 2.1-8.4; p-value < 0.001) and other (IRR: 4.6; 95% CI: 2.3-9.1; p-value < 0.001)) and CD4 cell count ≤ 200 cells/μL at HIV diagnosis (IRR: 3.8; 95% CI: 2.4-6.0; p-value < 0.001). In the multivariate analysis only African (IRR: 4.3; 95% CI: 2.6-7.3; p-value < 0.001) and Asian (IRR: 5.7; 95% CI: 3.2-10.1; p-value < 0.001) origin, route of HIV infection (heterosexual (IRR: 1.9; 95% CI: 1.0-3.5; p-value: 0.048), IDU (IRR: 3.0; 95% CI: 1.5-6.2; p-value: 0.003) and other (IRR: 2.4; 95% CI: 1.2-4.9; p-value: 0.020)) and CD4 cell count ≤ 200 cells/μL at HIV diagnosis (IRR: 1.6; 95% CI: 1.1-2.2; p-value: 0.005) preserved a statistically significant association with increased risk of TB diagnosis. After start of HAART the unadjusted and adjusted IRR for TB respectively were 0.04 (95% CI: 0.03-0.07; p-value < 0.001) and 0.05 (95% CI: 0.03 - 0.08; p-value < 0.001) compared to the time period before initiation of HAART (table 4).

**Table 4:** Risk factors for being diagnosed with TB in HIV infected patients in Denmark

Risk factor		Unadjusted analysis		Adjusted analysis	
		**Incidence rate ratio ** (95% CI*)	**p-value**	**Incidence rate ratio** (95% CI*)	**p-value**

Male gender		2.1 (1.5-3.0)	< 0.001	1.0 (0.6-1.5)	0.925
Age < 40 years		1.5 (1.0-2.3)	0.036	0.9 (0.6-1.4)	0.603
Year of HIV	1995-1996	1.0			1.0
Diagnosis	1997-1999	1.1 (0.6-1.9)	0.793	1.0 (0.6-1.6)	0.937
	2000-2007	1.0 (0.6-1.7)	0.896	0.9 (0.5-1.6)	0.684
Country of birth	Denmark	1.0		1.0	
	Europe	1.0 (0.4-2.9)	0.955	1.2 (0.4-3.4)	0.709
	Africa	4.7 (3.1-7.0)	< 0.001	4.3 (2.6-7.3)	< 0.001
	Asia	5.9 (3.5-9.8)	< 0.001	5.7 (3.2-10.1)	< 0.001
	Other	2.0 (0.7-5.7)	0.173	1.3 (0.5-3.9)	0.586
Route of	Homosexual	1.0		1.0	
Transmission	Heterosexual	3.9 (2.3-6.5)	< 0.001	1.9 (1.0-3.5)	0.048
	IDU	4.2 (2.1-8.4)	< 0.001	3.0 (1.5-6.2)	0.003
	Other	4.6 (2.3-9.1)	< 0.001	2.4 (1.2-4.9)	0.020
Viral load at HIV diagnosis, log_10 _copies/mL**	> 5.0	1.4 (0.8-2.2)	0.223	1.12 (0.8-1.6)	0.500
CD4 cell count at HIV diagnosis, cells/μL***	≤ 200	3.8 (2.4-6.0)	< 0.001	1.6 (1.1-2.2)	0.005
Diagnosed with alcohol abuse		1.3 (0.6-2.7)	0.447	1.9 (0.9-4.0)	0.106
Start of HAART (time-updated)		0.04 (0.03 - 0.07)	< 0.001	0.05 (0.03-0.08)	< 0.001

* CI: Confidence Interval.

** 673 patients has missing viral load at time of HIV diagnosis.

*** 532 patients had missing CD4 cell count at time of HIV diagnosis.

**Figure 1:**
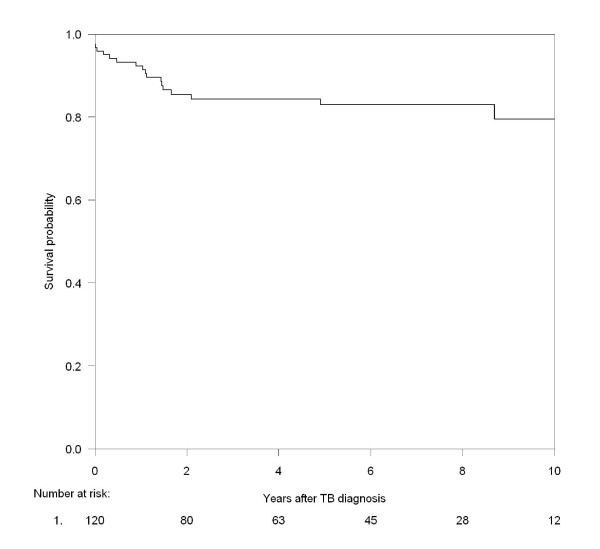
**Probability of survival after diagnosis of tuberculosis**.

Of the 120 TB patients, 19 died during the study period. Five deaths were recorded as related to TB, eight deaths were recorded as related to HIV and six died of other causes not related to HIV or TB or the cause of death was unknown. 16 of the 19 patients died within the first two years after HIV diagnosis. 9 (47%) of the 19 patients had a CD4 cell count < 200 cells/μl and 7 (37%) had a CD4 cell count < 50 cells/μl at time of death. The mortality was highest in the first two years after TB diagnosis with a two year probability of survival of 85% (95% CI; 78% - 92%), Figure 1. The five year probability of survival was 81% (95% CI; 74% - 89%).

## Discussion

In this nationwide, population-based cohort study, we found that the incidence of TB in HIV-infected individuals peaks during the first year after HIV diagnosis and decreased markedly after the introduction of HAART. African and Asian origin, low CD4 cell count at HIV diagnosis, lack of HAART and heterosexual and IDU route of HIV transmission were risk factors for being diagnosed with TB. To our knowledge this study is the first to present a comprehensive, nationwide analysis of incidence, risk factors of TB and prognosis of TB in HIV infected individuals. Further, the study design allowed long and nearly complete follow-up with the inclusion of only patients diagnosed with HIV in the study period after arrival to Denmark. Of importance our study population was incident concerning HIV and TB diagnosis.

The TB diagnosis was included in our analysis regardless of the patient being classified with presumptive or definite TB. Some patients may therefore have been misclassified leading to overestimation of the incidence. However, due to the complexity of diagnosing TB among HIV-positive individuals, presumptive TB, being clinical symptoms and/or paraclinical findings suggestive of TB, is by the majority considered sufficient for further measures and initiation of TB treatment [5,13]. Unfortunately our study does not include data on immune restitution inflammatory response (IRIS) which has been reported in up to 7-36% in HAART-naive HIV patients co-infected with TB [14].

Despite our large sample size and relatively long period of observation, only 120 cases of TB were diagnosed, which limits the strength of the multivariate analyses. To obtain an incident cohort of HIV and TB we excluded 233 patients diagnosed with HIV before arrival in Denmark. Of these patients, 6 were found to have TB. With regard to our findings on risk factors for TB, the omission of these patients may have altered our conclusions. The small number of deaths did not allow us to identify risk factors for death among the HIV patients with TB.

We chose to adjust for alcohol abuse using alcohol related discharge diagnoses as registered in the Danish Hospital Database, knowing that this is an incomplete measure giving rise to unmeasured and residual confounding.

We found a high incidence rate of TB among HIV positive patients in Denmark of 8.2/1,000 PYR in the period 1995-2007. The incidence of TB was remarkably high the first year after being diagnosed with HIV - irrespective of the time period studied. Hereafter the incidence rates declined steadily over time. Other studies from regions with low TB incidence have found similar incidence rates; Girardi *et al. *found an overall incidence of 4.7 cases per 1,000 PYR in Europe and North America in a study conducted in the HAART era [15]. In HIV patients from the United States, Markowitz *et al. *found an incidence of 7 TB cases per 1,000 PYR [16], a figure similar to that found in the EuroSIDA cohort, which estimated an incidence of 8/1,000 PYR [5].

Interestingly, the incidence of TB declined significantly with time after HIV-diagnosis, independently of the time periods (pre-, early or late- HAART). Within the first year of HIV-diagnosis we found an incidence of 39.9 TB cases per 1,000 PYFU, a number approaching that seen in Ethiopia and South Africa. In South Africa, however, the high incidence persists after 5 years, being 10-fold of that found in our study five years after HIV-diagnosis, probably due to high prevalence of latent TB infection and advanced immune suppression in a population where HAART is not widely implemented or by other co-morbidity [17,18]. Likewise, in our study the decline in incidence of TB with time after HIV diagnosis is most likely attributed to immunological restitution due to HAART. Initiation of HAART and immunological restitution may "unmask" TB as a part of IRIS which probably also partially explains the high incidence of TB in the first year after HIV diagnosis. Besides this, TB is the most frequent AIDS defining disease and in many African countries more than half of the TB patients are diagnosed with HIV when diagnosed with active TB. This bias may also contribute to the extremely high incidence rate of TB seen in connection with the HIV diagnosis and explain the rapid decline after diagnosis.

Concerning risk of a TB diagnosis, observational studies conducted both in low- and high-income countries have shown that the risk of TB has decreased 50-90% under HAART, yet not approaching that in the general population [2,18]. Our study supports this finding with a more than 90% decreased risk of TB after initiation of HAART. However, some subgroups still remain at a higher risk of developing TB than others. Findings similar to ours have been established in Brazil where baseline CD4 cell counts of ≤ 200 cells/μL and lack of HAART treatment both were independently associated with increased risk of TB [18]. In a study cohort from the United States, IDU, heterosexual contact, CD4 cell counts ≤ 100 cells/μL and lack of antiretroviral therapy were important risk factors for TB [16]. Not surprisingly patients from high TB burden countries are at greater risk of TB than others due to reactivation of latent TB [8]. This may explain the finding of a higher risk of TB among heterosexually HIV infected as this is the most likely route of HIV transmission for the majority of African and Asian immigrants [19,20].

The mortality was increased in the first two years after the TB diagnosis, presumably due to a high degree of immunodeficiency among the TB patients. The prognosis was good after the first two years. The five year probability of survival was comparable to that of other studies [17,18,21].

## Conclusions

We conclude that although the risk of TB has decreased after the introduction of HAART it is still high and substantially higher in the first year after HIV diagnosis and associated with African and Asian origin, low CD4 count and non-homosexual route of HIV infection. The mortality is high in this patient population in the first 2 years after TB diagnosis.

## Competing interests

Potential competing of interest: N Obel has received research funding from Roche, Bristol-Myers Squibb, Merck Sharp & Dohme, GlaxoSmithKline, Abbott, Boehringer Ingelheim, Janssen-Cilag and Swedish Orphan. F Engsig has received research funding from Merck Sharp & Dohme.

None of the other authors have competing interests.

## Authors' contributions

The authors contributions are the following: GAT (MD) and FNE (MD) contributed with study design, data collection, data analysis, interpretation of findings and writing of the manuscript. ABA (MD, Professor, DrMedSc) and PR (MD, PhD) contributed with study design, interpretation of findings and critical edit of the manuscript. ISJ (MD, DrMedSc), CSL (MD, DrMedSc) and BR (MD, PhD) contributed with data collection, study design, interpretation of findings and critical edit of the manuscript. NO (MD, DrMedSc) contributed with data collection, study design, critical review of data analyses, interpretation of findings and critical edit of the manuscript. All authors read and approved the final manuscript.

## Pre-publication history

The pre-publication history for this paper can be accessed here:

http://www.biomedcentral.com/1471-2466/11/26/prepub
